# Clonal Structure, Virulence Factor-encoding Genes and Antibiotic Resistance of *Escherichia coli*, Causing Urinary Tract Infections and Other Extraintestinal Infections in Humans in Spain and France during 2016

**DOI:** 10.3390/antibiotics9040161

**Published:** 2020-04-04

**Authors:** Saskia-Camille Flament-Simon, Marie-Hélène Nicolas-Chanoine, Vanesa García, Marion Duprilot, Noémie Mayer, María Pilar Alonso, Isidro García-Meniño, Jesús E. Blanco, Miguel Blanco, Jorge Blanco

**Affiliations:** 1 Laboratorio de Referencia de E. coli (LREC), Departamento de Microbioloxía e Parasitoloxía, Facultade de Veterinaria, Universidade de Santiago de Compostela (USC), 27002 Lugo, Spain; saskia.flament@usc.es (S.-C.F.-S.); vanesag.menendez@usc.es (V.G.); isidro.garcia@usc.es (I.G.-M.); jesuseulogio.blanco@usc.es (J.E.B.); miguel.blanco@usc.es (M.B.); 2 Instituto de Investigación Sanitaria de Santiago de Compostela (IDIS), 15706 Santiago de Compostela, Spain; Pilar.Alonso.Garcia@sergas.es; 3 Service de Microbiologie, Hôpital Beaujon, AP-HP, 92110 Clichy, France; mhnchanoine@gmail.com (M.-H.N.-C.); marion.duprilot@gmail.com (M.D.); noemie.mayer@aphp.fr (N.M.); 4 Université de Paris Diderot, IAME, UMR 1137, INSERM, F-75018 Paris, France; 5 Unidade de Microbioloxía, Hospital Universitario Lucus Augusti (HULA), 27003 Lugo, Spain

**Keywords:** *E. coli*, clonal structure, ST88, ST141, ST131, ST1193, virulence genes, resistance

## Abstract

*Escherichia coli* is the main pathogen responsible for extraintestinal infections. A total of 196 clinical *E. coli* consecutively isolated during 2016 in Spain (100 from Lucus Augusti hospital in Lugo) and France (96 from Beaujon hospital in Clichy) were characterized. Phylogroups, clonotypes, sequence types (STs), O:H serotypes, virulence factor (VF)-encoding genes and antibiotic resistance were determined. Approximately 10% of the infections were caused by ST131 isolates in both hospitals and approximately 60% of these infections were caused by isolates belonging to only 10 STs (ST10, ST12, ST58, ST69, ST73, ST88, ST95, ST127, ST131, ST141). ST88 isolates were frequent, especially in Spain, while ST141 isolates significantly predominated in France. The 23 ST131 isolates displayed four clonotypes: CH40-30, CH40-41, CH40-22 and CH40-298. Only 13 (6.6%) isolates were carriers of extended-spectrum beta-lactamase (ESBL) enzymes. However, 37.2% of the isolates were multidrug-resistant (MDR). Approximately 40% of the MDR isolates belonged to only four of the dominant clones (B2-CH40-30-ST131, B2-CH40-41-ST131, C-CH4-39-ST88 and D-CH35-27-ST69). Among the remaining MDR isolates, two isolates belonged to B2-CH14-64-ST1193, i.e., the new global emergent MDR clone. Moreover, a hybrid extraintestinal pathogenic *E.coli* (ExPEC)/enteroaggregative isolate belonging to the A-CH11-54-ST10 clone was identified.

## 1. Introduction

*Escherichia coli* is the leading cause of urinary tract (UTI) and bloodstream infections. Most infections like this are due to isolates of pathotypes known as extraintestinal pathogenic *E. coli* (ExPEC) or uropathogenic *E. coli* (UPEC) [1,2]. Numerous virulence genes have been associated with isolates causing extraintestinal infections, such as adhesins, toxins, siderophores and capsular antigens, that enable them to colonize host surfaces, capture available iron, injure host tissues and avoid host defense systems. 

The treatment of these infections has been seriously complicated by the appearance of multidrug-resistant (MDR) isolates and especially by the rapid dissemination of extended-spectrum β-lactamase-producing *E. coli* (ESBL-EC) [3,4,5].

There is an enormous diversity among *E. coli* isolates causing extraintestinal infections, however, epidemiological studies indicate that certain O:H serotypes and sequence types (STs) are more predominant and especially successful [6,7,8]. Twenty major STs (in order of highest to lowest prevalence: ST131, ST69, ST10, ST405, ST38, ST95, ST648, ST73, ST410, ST393, ST354, ST12, ST127, ST167, ST58, ST88, ST617, ST23, ST117 and ST1193) accounted for 85% of the *E. coli* isolates from 217 meta-analyzed studies (1995 and 2018), systematically reviewed by Manges et al. [8] However, most of the studies have been carried out on MDR and ESBL-producing isolates, but very few have been focused on any type of *E. coli* causing extraintestinal infections and, furthermore, on their clonal structure. Therefore, there is probably an overestimation of some STs and an underestimation of others. 

To our knowledge, the present study is the first one that is conducted, concomitantly, during a recent time period in two European countries, Spain and France, and provides data on the phylogroups, serotypes, clonal structure, virulence factor-encoding genes and antibiotic resistance displayed by all of the *E. coli* clinical isolates consecutively obtained.

## 2. Results

### 2.1. Phylogenetic Groups

**Figure 1:**
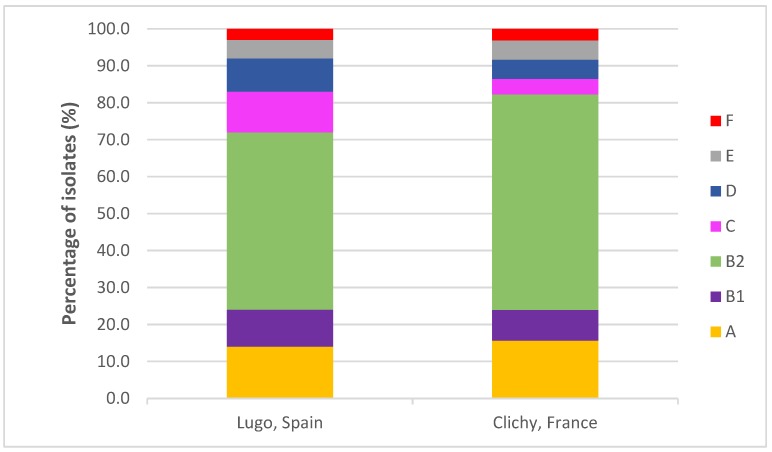
Comparison of the distribution of phylogenetic groups in the two hospitals.

The most frequent phylogenetic group in both hospitals was B2 (48%-Spain vs. 58.3%-France) followed by the other six phylogenetic groups: A (14% vs. 15.6%), B1 (10% vs. 8.3%), C (11% vs. 4.2%), D (9% vs. 5.2%), E (5% vs. 5.2%) and F (3% vs. 3.1%). Although we observed a higher prevalence of B2 isolates in the French hospital and C isolates in the Spanish hospital, the differences were not statistically significant (Table S1 and Figure 1).

### 2.2. Serotypes and Sequence Types

**Figure 2:**
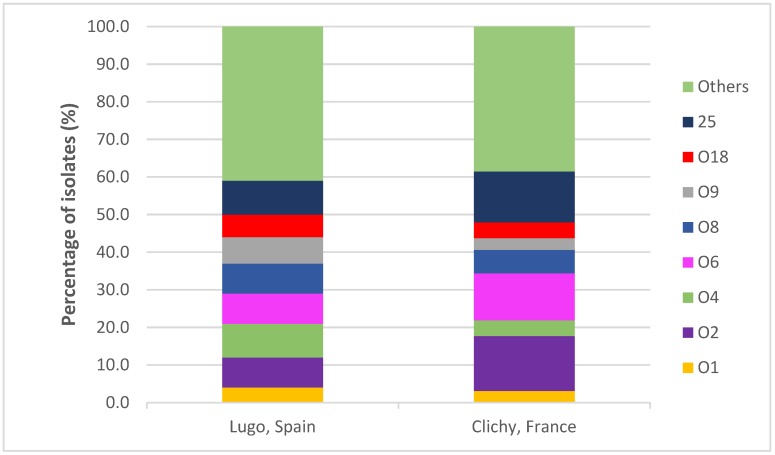
Comparison of the distribution of O serogroups in the two hospitals.

Forty O serogroups were found, but 118 (60.2%) of the 196 isolates belonged to one of the following eight serogroups: O1 (3.6%), O2 (11.2%), O4 (6.6%), O6 (10.2%), O8 (7.1%), O9 (5.1%), O18 (5.1%) and O25 (11.2%) (Figure 2). The isolates expressed 21 different H antigens, but 119 (60.7%) isolates showed only seven types of flagellar antigens: H1 (11.2%), H4 (23.5%), H5 (4.1%), H6 (7.7%), H7 (6.1%), H18 (5.1%) and H31 (3.1%) (Table 1). 

**Figure 3:**
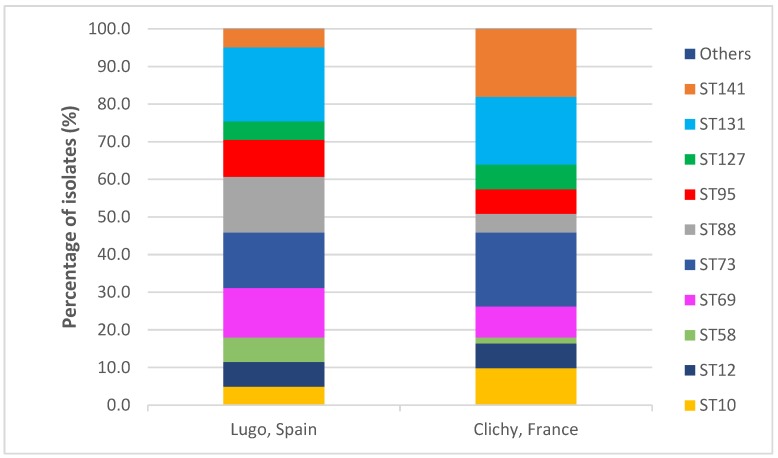
Comparison of the distribution of sequence types in the two hospitals.

Seventy-one STs were found among the 196 studied isolates, but 61% and 63.5% of Spanish and French isolates, respectively, belonged to one of the ten following STs: ST10 (3% vs. 6.3%), ST12 (4% vs. 4.2%), ST69 (8% vs. 5.2%), ST58 (4% vs. 1%), ST73 (9% vs. 12.5%), ST88 (9% vs. 3.1%), ST95 (6% vs. 4.2%), ST127 (3% vs. 4.2%), ST131 (12% vs. 11.5%) and ST141 (3% vs. 11.5%). Statistically significant differences between the two hospitals were observed only with respect to ST141 (P = 0.026) (Table S1 and Figure 3).

A strong association between certain serotypes and STs was observed. Thus, the 11 isolates of serotype O2:H6 were ST141, the 12 O6:H1 isolates were ST73, 5 of the 6 O6:HNM isolates were ST127, 5 of the 6 O8:H4 isolates were ST88, 3 of the 4 O9:H4 isolates were ST88, and 17 of the 21 O25:H4 isolates were ST131 (Table 1).

### 2.3. Clones

A total of 107 clones (defined by the association of phylogroup, clonotype and ST) were identified among the 196 isolates, with 26 of them including at least two isolates and only nine at least four isolates: B2-CH13-106-ST12 (4 isolates), B2-CH24-10-ST73 (6), B2-CH24-103-ST73 (6), B2-CH38-15-ST95 (4), B2-CH40-30-ST131 (13), B2-CH40-41-ST131 (6), B2-CH52-5-ST141 (11), C-CH4-39-ST88 (10) and D-CH35-27-ST69 (13) (Table 1). Only statistically significant differences were observed with respect to clone B2-CH52-5-ST141, which was more prevalent in the French hospital (P = 0.018). 

### 2.4. Virulence Factor (VF)-Encoding Genes

Of the 31 VF-encoding genes analyzed, four (*fimH*, *fyuA*, *chuA*, *ompT*) were detected in more than 60% of the 196 isolates and 11 (*papAH*, *papC*, *papEF*, *yfcV*, *vat*, *iucD*, *iutA*, *iroN*, *traT*, *malX*, *usp*) in at least 40%. In contrast, five VF-encoding genes (*afa/draBC*, *cdtB*, *tsh*, *kpsM II-K2*, *kpsM III*) were found in less than 10% of the isolates (Table 2). 

**Figure 4:**
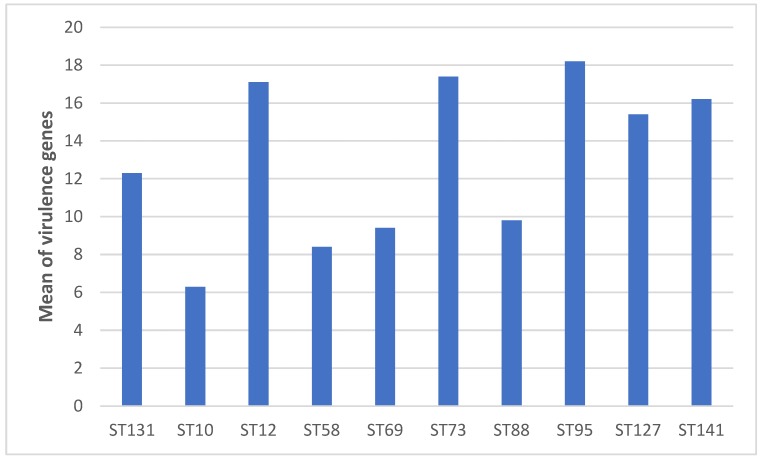
Mean of VF-encoding genes within dominant sequence types.

There was a strong correlation between VF-encoding gene profiles and STs. A higher mean of VF-encoding gene score was observed in the isolates belonging to the following dominant B2- phylogenetic group STs (ST12, 17.1; ST73, 17.4; ST95, 18.2; ST127, 15.4; ST131, 12.3 and ST141, 16.2), compared with the dominant STs belonging to phylogroups A (ST10, 6.3), B1 (ST58, 8.4), C (ST88, 9.8) and D (ST69, 9.4) (Table 2 and Figure 4).

Of the 196 isolates, 61.7% were presumptively classified as extraintestinal pathogenic *E. coli* (ExPEC) and 54.1% as uropathogenic *E. coli* (UPEC). All ST12, ST73, ST95, ST127 and ST141 isolates and the majority of ST131 isolates were classified as UPEC. In contrast, none of the ST10, ST58, ST69 and ST88 isolates presented the virulence markers necessary to be classified as UPEC (Table 2). 

### 2.5. Clonotypes, Clades, Subclades, Clusters and virotypes of ST131 Isolates

The 23 isolates of the dominant ST in both countries, i.e., ST131, were distributed in four clonotypes: CH40-30 (5 Spanish isolates vs. 8 French isolates), CH40-41 (5 vs. 1), CH40-22 (1 vs. 2) and CH40-298 (1 vs. 0) (Table S1).

**Figure 5:**
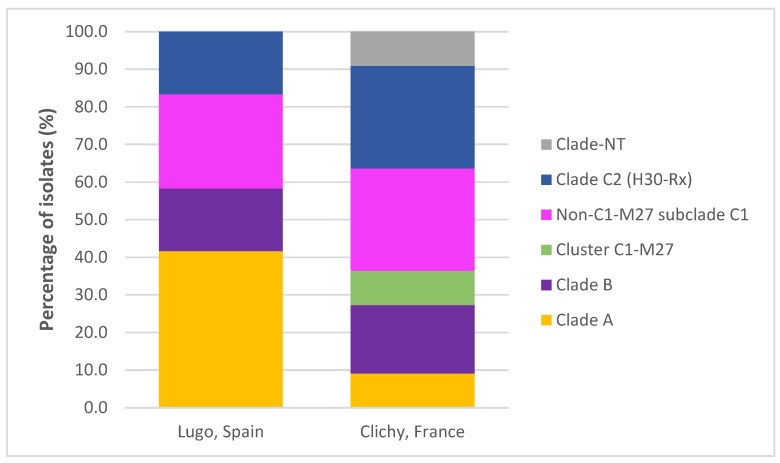
Comparison of the distribution of sequence type 131 (ST131) clades and clusters in the two hospitals.

Isolates of clade A and non-C1-M27 subclade C1 were the most commonly detected (6 isolates for each), followed by those of subclade C2 (also known as subclone *H*30Rx) (5 isolates), clade B (4 isolates) and cluster C1-M27 (1 isolate) (Table S1 and Figure 5). 

The previously determined ST131 virotypes were identified in 17 of the 23 ST131 isolates (Table S1), among which virotypes A (3 isolates), C2 (3 isolates) and C3 (3 isolates) were most prevalent. The virotypes of six strains could not be determined, since they showed new combinations of virulence genes not included in the classification scheme used (Table S1).

### 2.6. Antimicrobial Resistance

The prevalence of resistance to ampicillin, doxycycline, nalidixic acid, ciprofloxacin and trimethoprim-sulfamethoxazole was >20%. In contrast, no isolates resistant to amikacin, colistin, fosfomycin or imipenem were detected (Table 3). 

**Figure 6:**
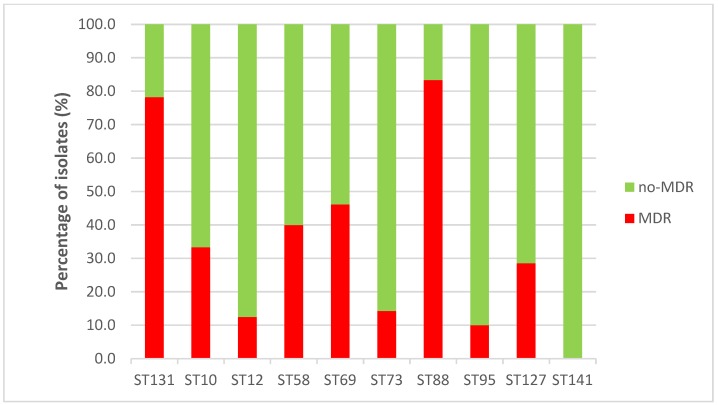
Multidrug resistance (MDR) within dominant sequence types.

Only 13 (6.6%) isolates produced an ESBL enzyme: CTX-M-1 (4 isolates), CTX-M-14 (3), CTX-M-15 (3), CTX-M-27 (1), CTX-M-55 (1) and CTX-M-32 (1) (Table S2). However, 73 (37.2%) of the 196 analyzed isolates were classified as MDR. MDR was especially associated with ST88 (83.3%) and ST131 (78.3%) isolates, but an important percentage of ST58 (40%) and ST69 (42.6%) isolates were also classified as MDR (Figure 6). The MDR differences between ST88 and ST131 with respect to ST10 (33.3%), ST12 (12.5%), ST73 (14.3%), ST95 (10%), ST127 (28.6%) and ST141 (0%) were statistically significant. The new global emergent MDR clonal group, i.e., ST1193 [9], [10], was displayed by two isolates, one from Spain and the other one from France (Table 1). Among the 73 MDR isolates, 31 (42.5%) belonged to only four clones: B2-CH40-30-ST131 (12 isolates), B2-CH40-41-ST131 (4), C-CH4-39-ST88 (9) and D-CH35-27-ST69 (6) (Table 1). Clone B2-CH40-30-ST131 was also the most prevalent among the ESBL-producing isolates (4 of 13 isolates) (Table S2).

Fourteen (*papAH*, *papC*, *sfa/focDE*, *yfcV*, *cnf1*, *hlyA*, *vat*, *iroN*, *chuA*, *neuC-K1*, *ibeA*, *malX*, *usp* and *ompT*) of the 31 analyzed VF-encoding genes were found to be associated with isolates that did not show multidrug resistance, while only the *traT* gene was found to be associated with MDR isolates (Table S3). 

### 2.7. Hybrid Pathotypes 

In one of the 196 isolates that displayed serotype O153:HNT, the three (*aatA*, *aaiC* and *aggR*) genes specific for EAEC were detected. This hybrid ExPEC/EAEC isolate, which belonged to the A-CH11-54-ST10 clone, harbored 14 additional VF-encoding genes specific for ExPEC (*fimH*, fimAv_MT78_, *papAH*, *papC*, *papEF*, *afa/draBC*, *sat, hlyA*, *iucD*, *iutA*, *fyuA*, *kpsM II-K5*, *traT*, *ompT*), and was resistant to ampicillin and doxycycline. None of the genes specific for the different diarrheagenic *E. coli* pathovars was detected in the remaining 195 isolates. 

## 3. Discussion

To our knowledge, this is the first study conducted in Spain in which the clonal structure of extraintestinal pathogenic *E. coli* was analyzed from clinical *E. coli* non-redundant and consecutively isolated. Indeed, the previous Spanish studies focused on selected clinical isolates. Thus, Blanco et al. [11] evaluated the incidence of only three high-risk clones among 500 consecutively collected *E. coli* isolates, causing extraintestinal infections in five Spanish hospitals in 2009. They found that ST131 accounted for 12%, ST393 for 3% and ST69 for 4%, and these three clones accounted for 30% of the MDR isolates. In the present study, ST131 was one of the most prevalent ST in the enrolled Spanish hospital and accounted for 12%. This result strongly suggests that the ST131 rate has remained stable between 2009 and 2016 in Spain. The ST69 rate seems to have increased as this ST accounted for 8% in 2016, compared to 4% in 2009. Inversely, the non-detection of any ST393 isolate in the current study seems to indicate that its rate has declined dramatically in Spain between 2009 and 2016. Oteo et al. [12] analyzed the ST distribution among OXA-1-producing *E. coli* isolates resistant to amoxicillin-clavulanate collected from clinical samples (>70% from urine), from seven Spanish hospitals in 2010 and found that ST88 (37.3%) and ST131 (32.8%) were the most prevalent STs in this *E. coli* subgroup. Interestingly, we found in the present study that ST88 (9%) was one of the most common STs after ST131 in Spain but not in France (3.1%).

In France, van der Mee-Marquet et al. [13] studied the genetic diversity of 412 *E. coli* bloodstream isolates recovered during 2014 and found 12 major ST complexes (STCs 10, 12, 14, 23, 31, 69, 73, 92, 95, 131, 141 and 155), containing 77.9% of the collected isolates. Clermont et al. [14] analyzed 243 bacteremia isolates obtained in 2010 in the Paris area and found four most prevalent STCs: STC95 (14.0%), STC73 (13.2%), STC131 (8.6%) and STC69 (7.8%). La Combe et al. [15] analyzed 260 respiratory tract isolates obtained from patients who had pneumonia and were hospitalized between 2012 and 2014 in intensive care units located throughout France. The most commonly identified lineages were STC73 (15.4%), STC131 (9.2%), STC69 (7.7%), STC141 (6.2%), STC127 (6.2%) and STC95 (5.4%). They compared this STC distribution to that observed in bacteremia and commensal isolates and noted that STC127 and STC141 were overrepresented and STC95 underrepresented in pneumonia isolates compared with bacteremia isolates. Brisse et al. [16] compared the phylogenetic diversity of 152 CTX-M-producing and 152 non-ESBL-producing clinical *E. coli* isolates, obtained between 2008 and 2009 from ten hospitals located in the Paris area. The five most prevalent STs were distributed differently between CTX-M-producing and non-ESBL-producing *E. coli* isolates: ST131 (36.2% vs. 9.9%), ST10 (8.6% vs. 5.9%), ST73 (0% vs. 10.5%), ST95 (0% vs. 5.3%) and ST141 (0% vs. 4.6%). In the present study, the most prevalent STs in the enrolled French hospital were ST73 (12.5%), ST131 and ST141 (11.5% for each), ST10 (6.3%), ST69 (5.2%), ST12, ST95 and ST127 (4.2% for each). The comparative analysis of the present study and the four previously published studies shows that the distribution of the most prevalent STs appears to have been stable in France during the period-time covered by the five studies (2008-2016). However, our study highlighted that ST141, which was already present in 2008 [17], has not only persisted in any type of source, except for blood, but has increased to become as prevalent as ST131, although it does not belong to the MDR-ST group. 

The clonal structure of different collections of *E. coli* clinical isolates has also been investigated in other countries of the European continent. Thus, different studies were conducted in UK, focusing either on UTI isolates (2007–2009) [18] or bacteremia isolates (2001–2012) [19,20,21]. Independently of the sample source and the location of the enrolled centers, the distribution of the predominant STs was the same in UK, with some variations in terms of frequency. Thus, ST73 varied from 16.6% to 18.6%, ST131 from 12% to 16.8%, ST69 from 5.4% to 10.5%, ST95 from 6.3% to 10.6% and ST12 from 4.4% to 4.6%. ST10 (4.3%) and ST127 (3.6%) were identified as prevalent ST among only the UTI isolates and ST12 only among bacteremia isolates. Kallonen et al. [21] highlighted that after the emergence of ST69 (2002) and ST131 (2003) and their spread, a new equilibrium of *E. coli* populations was observed, resulting in a relative stability of the major STs. The notable difference between the UK studies and ours is the absence of ST88 and ST141 among the predominant STs in UK, whereas the rate of ST88 was high in Spain (9%) and that of ST141 in France (11.5%). In contrast, both ST88 and ST141 were identified in Germany among the prevalent STs and each accounted for 4.2% of 265 UTI isolates collected between 2004 and 2006 [22]. ST141 was also identified among 44 *E. coli* isolated from UTIs in Switzerland during 2016. It accounted for 11.4% of the isolates, whereas ST131 and ST69 accounted, each, for 13.6%, and ST73 for 6.8% [23]. These features suggest that the clonal structure of *E. coli* in Germany and Switzerland seems to be closer to that of Spain and France than to that of UK. 

Some studies were also conducted in North America. In Canada, Fibke et al. [24] analyzed the genome of *E. coli* isolates responsible for UTI, in 385 women between 2012 and 2015. The major STs included ST95 (18.4%), ST73 (10.1%), ST127 (9.1%), ST131 (8.8%) and ST69 (7.5%). Among the studies performed in the USA on UTI isolates, different ST distributions were observed. Thus, Banerjee et al. [25] studied 299 clinical extraintestinal *E. coli* isolates (90% from UTIs) obtained during 2011 in Minnesota, and identified the five most prevalent STs, consisting of ST131 (27%), ST95 (11%), ST73 (8%), ST127 (6%) and ST69 (5%). On their side, Yamaji et al. [26], who studied 233 *E. coli* isolated from UTIs in California during 2016 and 2017, and 225 isolates collected similarly between 1999 and 2000, showed that the ST131 isolates were less frequently identified than the ST95, ST127, ST73, and ST69 isolates in these two periods. Similar to UTI isolates, bacteremia isolates were differently distributed in ST according to the studies. Thus, Adams-Sapper et al. [27], analyzing 220 bloodstream isolates obtained in San Francisco between 2007 and 2010, found only five prevalent STs and clonal complexes, accounting for 65% of the isolates. They comprised ST131 (23%), ST95 (18%), STC73 (8%), ST69 (9%) and STC12 (6%). On their side, Cole et al. [28], studying 43 bacteremia *E. coli* isolates from newborns obtained between 2006 and 2016 in Oklahoma, found that ST95 was the most prevalent ST (11.3%) followed by ST131 (9.2%) and ST1193 (3.7%). Cole et al. [28] noted that ST95 and ST131 isolates were present throughout the studied years, while ST1193 was only seen in the recent years. Overall, neither ST88 nor ST141 belong to the most prevalent clones in North America. In North America, ST95 is more prevalent than ST73, while, in Europe, the opposite is true.

Similar to Cole et al. [28], we recently identified, i.e. in 2016, the global emergent MDR ST1193. To our knowledge, it is the first identification of this clone in Spain and the second in France. Indeed, Birgy et al. [29] found that among 218 ESBL-producing *E. coli* causing febrile UTI in children between 2014–2017, ST1193 was one of the most prevalent clones during the most recent period of the study. 

ST1193 seems to be more prevalent in Asia than in Europe and the USA. Indeed, Chen et al. [30], who characterized 100 bloodstream *E. coli* isolates from Zhejiang (China) in 2015 showed that, among the most prevalent clones, ST131 (15.3%) was followed by ST1193 (7.1%), then, by ST95 (5.9%) and ST69 (5.9%). This ST distribution suggests that strong antibiotic pressure has displaced the frequently antimicrobial susceptible STs among blood *E. coli* isolates, such as ST73 and ST95. However, in China, ST95 isolates have not been fully displaced, since some isolates have acquired plasmids encoding ESBL enzymes.

To our knowledge, the analysis of the clonotypes (analysis of the *fumC* and *fimH* alleles: CH), in addition to the phylogroup and ST types, allowing clone determination, was only carried out by Tchesnokova et al. [31] in the USA and Matsumura et al. [32] in Japan. Tchesnokova et al. [31] found 222 CH clonotypes among 1518 *E. coli* isolates (93% from UTIs), recovered between 2010 and 2011. Matsumura et al. [32] found 103 clonotypes among 329 *E. coli* (65% from UTIs and 11.9% from bacteremia), collected from 10 Japanese hospitals in 2014. In our study, 107 clonotypes were identified among 196 isolates, which suggests a higher diversity of clones in our study than in the American and Japanese studies. However, the most remarkable difference between the three studies is the distribution of the most prevalent STs among the MDR isolates: B2-CH40-30-ST131 and D-CH35-27-ST69 clones in the USA, B2-CH40-30-ST131 clone in Japan, and B2-CH40-30-ST131, B2-CH40-41-ST131, C-CH4-39-ST88 and D-CH35-27-ST69 clones in Spain and France. Consequently, it can be noted that ST88 is not a predominant ST in Japan, like in UK and the USA.

Fibke et al. [24] provided data on the *fimH* alleles displayed by the 34 Canadian ST131, that they identified among UTI isolates: *fimH*22 (20.6%), *fimH*27 (5.9%), *fimH*30 (23.5%) and *fimH*41 (44.1%). In our study, the distribution of the *fimH* alleles among our 23 ST131 isolates was very different: *fimH*22 (13.0%), *fimH*30 (56.5%), *fimH*41 (26.1%) and *fimH*298 (4.3%). Kallonen et al. [21], based on single nucleotide polymorphisms (SNPs), identified eight clades among English ST73 isolates associated with different O:H serotypes. Using the *fimH* allele type, we identified six *fimH* alleles among our 23 ST73 isolates (*fimH* 10, 12, 13, 27, 30, 32 and 103), that defined six clones, among which B2-CH24-10-ST73 and B2-CH24-103-ST73 clones predominated. Gordon et al. [33] studied the phylogeny of 200 STC95 isolates from humans living in France, Australia and the USA. The SNPs analysis revealed five main clusters associated to different *fimH* alleles and serotypes. In the present study, we have also identified five clones among our 10 ST95 isolates with different *fimH* alleles (*fimH* 15, 27, 30, 41 and 54) and serotypes (O1:H7, O2:H4, O2:H5, O2:H7, O18:H7, O25:H4 and O45:H7). Here, the most common clone was the B2-CH38-15-ST95, displaying serotypes O2:H5 (1 isolate), O2:H7 (1), O18:H7 (2).

Taking into consideration that Gati et al. [17] identified hybrid shigatoxin-producing *E. coli* /UPEC isolates among ST141 isolates, we searched for these hybrid isolates among our 14 ST141 isolates, but no hybrid isolates were detected. However, we detected a French ExPEC isolate of serotype O153:HNT, that carried three VFs-encoding genes (*aatA*, *aaiC* and *aggR*), specific for EAEC. This hybrid belonged to A-CH11-54-ST10 clone and harbored 14 VFs typically found in ExPEC. Interestingly, Olesen et al. [34] found that the EAEC isolates of serotype O78:H80 and ST10 were responsible for an outbreak of UTI in Denmark. On their side, Abe et al. [35] and Lara et al. [36] found some UPEC isolates with properties of EAEC in Brazil, including hybrid UPEC/EAEC ST69, ST73 and ST131 isolates. 

## 4. Materials and Methods 

### 4.1. E. coli isolates 

A total of 196 non-duplicate (one isolate per patient) *E. coli* consecutively isolated during 2016 in Spain (100 from Lucus Augusti hospital in Lugo) and France (96 from Beaujon hospital in Clichy) were characterized. These two collections of isolates came from different clinical samples: 146 isolates from urine, 22 from blood, five from bile, three from ascitic fluid, six from abscesses and 14 from various other sources.

### 4.2. Phylogenetic Grouping, Serotyping, MLST, CH Typing and Identification of ST131 Clades and Subclades

- Assignment to the main phylogroups (A, B1, B2, C, D, E, F) was based on the protocol of Clermont et al. [37]
- The determination of O and H antigens was carried out using the method previously described by Guinée et al. [38], with all available O (O1 to O181) and H (H1 to H56) antisera. Isolates that did not react with any antisera were classified as O non-typeable (ONT) or HNT and those that were non-motile were denoted as HNM.
- The sequence types (STs) were established following the multilocus sequence typing (MLST) scheme of Achtman by gene amplification and sequencing of the seven housekeeping genes *(adk*, *fum*C, *gyr*B, *icd*, *mdh*, *pur*A, and *rec*A), according to the protocol and primers specified at the *E. coli* MLST web site (http://mlst.warwick.ac.uk/mlst/dbs/Ecoli) [39].
- Clonotype identification was determined by *fumC* and *fimH* (CH) sequencing [31,40].
- The ST131 clades (A, B, C), subclade C2 (also known as subclone *H*30Rx) and subclade C1 and cluster C1-M27 were established by PCR [41].

### 4.3. Virulence Genotyping 

Virulence factor (VF)-encoding genes of *E. coli* causing extraintestinal infections were screened by PCR [5] and the virotypes (A to F) of the ST131 isolates were established according to the scheme described by Dahbi et al. (2014) [42]. The virulence gene score was the number of extraintestinal virulence-associated genes detected. The isolates were designed presumptively as extraintestinal pathogenic *E. coli* (ExPEC) if positive for ≥2 of 5 markers, including *papAH* and/or *papC*, *sfa/focDE*, *afa/draBC*, *kpsM II*, and *iutA* [2], and as uropathogenic *E. coli* (UPEC) if positive for ≥3 of 4 markers, including *chuA*, *fyuA*, *vat*, and *yfcV* [1]. 

For detecting hybrid pathotypes, the 196 clinical isolates were also examined for ten VF-encoding genes, specific for pathotypes of diarrheagenic *E. coli* (DEC): typical and atypical enteropathogenic *E. coli* (tEPEC and aEPEC) (*eae* and *bfpA* genes), enteroinvasive *E. coli* (EIEC) (*ipaH* gene), enterotoxigenic *E. coli* (ETEC) (*eltA* and *est* genes), Shiga toxin-producing *E. coli* (STEC) (*stx*_1_ and *stx*_2_ genes) and enteroaggregative *E. coli* (EAEC) (*aatA*, *aaiC* and *aggR* genes) [43].

### 4.4. Antimicrobial Susceptibility and ESBL Typing

Antimicrobial susceptibility was determined by minimal inhibitory concentrations (MICs) and/ or disc diffusion. Resistance was interpreted based on the recommended breakpoints of the CLSI [44]. Fifteen classes of antimicrobial agents were analyzed: penicillins (ampicillin), penicillins and β-lactamase inhibitors (amoxicillin-clavulanic acid), non-extended spectrum cephalosporins 1^st^ and 2^nd^ generation cephalosporins (cefazolin and cefuroxime), extended-spectrum cephalosporins 3^rd^ and 4^th^ cephalosporins (cefotaxime, ceftazidime and cefepime), cephamycins (cefoxitin), monobactams (aztreonam), carbapenems (imipenem), aminoglycosides (gentamicin, tobramycin, amikacin), tetracyclines (doxycycline), phenicols (chloramphenicol), nitrofurans (nitrofurantoin), quinolones (nalidixic acid and ciprofloxacin), folate pathway inhibitors (trimethoprim-sulphamethoxazole), phosphonic acids (fosfomycin) and polymyxins (colistin). *E. coli* multidrug resistant (MDR) was defined as resistance to one or more agents in three or more classes of tested drugs [45].

Genetic identification of ESBL types was carried out by PCR followed by amplicon sequencing [46,47]. 

### 4.5. Statistical Analysis

All the *P* values were calculated using the Fisher’s exact test, except for the comparison of the means that was performed using the one-way ANOVA test. *P* values <0.05 were considered statistically significant. 

## 5. Conclusions

We concluded that approximately 10% of the extraintestinal *E. coli* infections that had occurred in 2016 in the two studied hospitals were caused by ST131 isolates, and approximately 60% of these infections were caused by isolates belonging to only 10 STs (ST10, ST12, ST58, ST69, ST73, ST88, ST95, ST127, ST131, ST141). ST88 and ST141 isolates were particularly frequent in the Spanish and French hospitals, respectively, while, so far, these two STs are absent among the dominant STs in UK, the USA and Asia but present in Germany and Switzerland. Our results confirm that, in Europe, ST73 is much more prevalent than ST95, while in North America, it is the contrary. The majority of ST12, ST73, ST95 and ST141 isolates were susceptible to most antibiotics, indicating that MDR was not the reason for their success. The results of the present study support the idea that their success is mainly due to the high number of VF-encoding genes that they possess. This study also shows that among the MDR isolates, four clones are predominant, especially: B2-CH40-30-ST131, B2-CH40-41-ST131, C-CH4-39-ST88 and D-CH35-27-ST69. Clone B2-CH40-30-ST131 was also the most prevalent clone among the ESBL-producing isolates. Finally, this study confirmed the presence of the new MDR global emergent ST1193, in France and in Spain. All these results suggest that surveillance of the clonal structure and antibiotic susceptibility of ExPEC is required at both local and global levels, notably to evaluate the evolutive impact of the antibiotic overuse on one of the most important human bacterial pathogens.

## Figures and Tables

**Table 1:** Clones, serotypes, and extraintestinal pathogenic *E. coli* (ExPEC), uropathogenic *E. coli* (UPEC), and multidrug resistant (MDR) status of the 196 isolates.

Clone (Number of Isolates from Spain and France) ^a,b^	Serotype (Number of Isolates)	ExPEC Status	UPEC Status	MDR Status
A-CH11-54-ST10 (1/2)	O48:HNM (1), O133:H40 (1), O153:HNT (1)	1	0	0
**B1-CH4-27-ST58** (2/1)	O8:HNM (1), O9:H4 (1), O9:H25 (1)	0	0	1
B2-CH13-27-ST12 (1/1)	O4:H5 (1), O4:HNM (1)	2	2	0
B2-CH13-106-ST12 (2/2)	O4:H1 (3), O4:HNM (1)	4	4	0
**B2-CH24-10-ST73** (2/4)	O2:H1 (1), O2:HNM (1), O6:H1 (3), O6:HNM (1)	6	6	1
B2-CH24-12-ST73 (2/1)	O6:H1 (1), O25:H1 (1), O25:H4 (1)	3	3	0
**B2-CH24-30-ST73** (0/3)	O6:H1 (3)	3	3	1
**B2-CH24-103-ST73** (4/2)	O6:H1 (4), O22:H1 (1), O22:HNM (1)	6	6	1
B2-CH38-15-ST95 (2/2)	O2:H5 (1), O2:H7 (1), O18:H7 (2)	4	4	0
**B2-CH38-27-ST95** (1/1)	O2:H4 (1), O25:H4 (1)	2	2	1
B2-CH38-41-ST95 (1/1)	O1:H7 (2)	2	2	0
**B2-CH14-2-ST127** (1/2)	O6:H31 (1), O6:HNM (2)	3	3	1
B2-CH14-136-ST127 (1/1)	O6:H31 (1), O6:HNM (1)	2	2	0
**B2-CH40-22-ST131** (1/2)	O25:H4 (3)	3	3	2
**B2-CH40-30-ST131** (5/8)	O25:H4 (13)	8	13	12
**B2-CH40-41-ST131** (5/1)	O4:H5 (2), O4:HNM (1) O12:HNM (1), ONT:H5 (2)	3	3	4
B2-CH52-5-ST141 (3/8)	O2:H6 (8), O14:HNM (1), ONT:H4 (1), ONT:H6 (1)	11	11	0
B2-CH52-14-ST141 (0/2)	O2:H6 (2)	2	2	0
B2-CH103-9-ST372 (1/1)	O18:H31 (2)	0	2	0
**B2-CH14-64-ST1193** (1/1)	O75:HNM (1), ONT:HNM (1)	1	2	2
**C-CH4-39-ST88** (9/1)	O8:H4 (5), O9:H4 (3), O9:HNM (1), ONT:H4 (1)	5	0	9
**D-CH35-27-ST69** (8/5)	O15:H18 (2), O15:HNM (1), O18:HNM (1), O44:H18 (2), O77:HNM (1), O106:H4 (2), O175:HNM (1), ONT:H18 (3)	9	0	6
**E-CH100-96-ST362** (2/1)	O7:H6 (2), O21:HNT (1)	1	0	3
**E-CH37-27-ST405** (1/1)	O18:H6 (1), O102:H4 (1)	1	0	1
F-CH32-41-ST59 (3/0)	O1:H7 (3)	3	3	0
**F-CH4-58-ST648** (0/2)	O25:H4 (1), ONT:H42 (1)	2	1	1

**^a^** Clones represented by a single isolate: A-CH11-23-ST10 (ONT:HNM) (F), A-CH11-27-ST10 (O14:HNM) (F), A-CH11-30-ST10 (O101:H4) (S), A-CH11-43-ST10 (O8:H4) (S), A-CH11-137-ST10 (O65:H10) (F), A-CH11-168-ST10 (O10:HNM) (F), A-CH11-54-ST34 (O9:H1) (S), A-CH11-54-ST44 (ONT:H49) (S), A-CH11-41-ST93 (O14:HNM) (F), A-CH11-NT-ST93 (O25:H4) (S), A-CH11-NEG-ST93 (O51:H52) (S), A-CH11-54-ST167 (O101:H21) (S), A-CH99-54-ST361 (O9:HNM) (S), A-CH4-34-ST399 (O126:H27) (F), A-CH107-233-ST401 (O21:H25) (S), A-CH7-53-ST540 (O14:HNM) (F), A-CH11-54-ST744 (ONT:H9) (S), A-CH7-86-ST746 (O171:HNT) (S), A-CH7-0-ST1139 (O9:HNM) (F), A-CH11-25-ST1141 (O73:H4) (S), A-CH4-NEG-ST1284 (O101:HNM) (F), A-CH4-0-ST2795 (O21:HNT) (F), A-CH11-43-ST3596 (O8:HNM) (F), A-CH11-27-ST3877 (O38:HNM) (F), A-CH7-54-ST new 1-540 Like (O9:HNM) (F), A-CH27-23-ST new 2-437 like (O98:HNM) (S), B1-CH4-25-ST17 (O4:H2) (S), B1-CH4-24-ST58 (ONT:H21) (S), B1-CH4-25-ST58 (O75:H20) (S), B1-CH41-86-ST101 (O103:H21) (S), B1-CH4-NEG-ST155 (ONT:HNM) (F), B1-CH8-31-ST210 (O155:H19) (S), B1-CH6-31-ST448 (O148:H8) (F), B1-CH6-34-ST448 (ONT:H8) (S), B1-CH6-35-ST448 (O11:H10) (S), B1-CH6-31-ST453 (O18:HNM) (F), B1-CH4-32-ST767 (ONT:H9) (S), B1-CH6-32-ST847 (ONT:H2) (F), B1-CH4-30-ST2025 (O8:HNM) (F), B1-CH4-NT-ST2077 (ONT:H2) (F), B1-CH4-NT-ST new 3-1071 like (O8:H8) (F), B2-CH13-6-ST12 (O4:H5) (S), B2-CH13-223-ST12 (O4:H5) (F), B2-CH14-27-ST14 (O18:HNM) (S), B2-CH14-NT-ST14 (O18:HNM) (F), B2-CH24-13-ST73 (O2:H1,12) (F), B2-CH24-27-ST73 (O6:H1) (F), B2-CH24-32-ST73 (O2:H1) (S), B2-CH24-NT-ST80 (O75:HNM) (F), B2-CH38-30-ST95 (ONT:H4) (S), B2-CH38-54-ST95 (O45:H7) (S), B2-CH24-2-ST104 (O22:H1) (S), B2-CH14-180-ST127 (O6:HNM) (F), B2-CH14-224-ST127 (O6:HNM) (S), B2-CH40-298-ST131 (O25:H4) (S), B2-CH52-76-ST141 (O2:H6) (F), B2-CH43-27-ST144 (O135:H4) (F), B2-CH14-27-ST404 (O75:HNM) (S), B2-CH38-92-ST421 (O1:H7) (F), B2-CH40-22-ST428 (O106:H4) (F), B2-CH40-21-ST555 (ONT:H4) (F), B2-CH43-13-ST567 (O83:HNM) (S), B2-CH38-5-ST569 (O46:H31) (S), B2-CH108-75-ST636 (O83:H7) (S), B2-CH38-18-ST1231 (O18:H7) (S), B2-CH319-197-ST2015 (O2:H14) (F), B2-CH40-22-ST2556 (ONT:H4) (F), B2-CH43-197-ST2558 (O2:H14) (S), B2-CH13-5-ST3352 (O4:HNM) (S), C-CH4-32-ST23 (O5:H31) (F), C-CH4-27-ST88 (O8:H19) (F), C-CH4-NT-ST88 (O8:HNM) (F), C-CH4-27-ST new 4-88-like (O8:H40) (S), C-CH4-24-ST410 (O8:H9) (S), D-CH35-47-ST106 (O77:H18) (S), E-CH26-65-ST38 (O1:H15) (F), E-CH31-54-ST57 (O114:H10) (S), E-CH26-270-ST115 (O2:H9) (S), E-CH26-27-ST963 (O2:H18) (F), E-CH26-5-ST new 5 (ONT:H18) (F), F-CH45-97-ST117 (ONT:H4) (F). ^**b**^ Bold highlights those clones that presented at least one MDR isolate. S = Spain. F = France.

**Table 2:** Virulence factor (VF)-encoding genes observed from the 196 isolates and the isolates included in the 10 most frequent sequence types.

VF Gene	Number (%) of Isolates										
	Total (*n* = 196)	B2 ST131 (*n* = 23)	A ST10 (*n* = 9)	B2 ST12 (*n* = 8)	B1 ST58 (*n* = 5)	D ST69 (*n* = 13)	B2 ST73 (*n* = 21)	C ST88 (*n* = 12)	B2 ST95 (*n* = 10)	B2 ST127 (*n* = 7)	B2 ST141 (*n* = 14)
**Adhesins**											
*fimH*	**193 (98.5)**	**23**	**9**	**8**	**5**	**13**	**21**	**12**	**10**	**7**	**14**
*fimAv_MT78_*	27 (13.8)	0	4	0	0	0	0	**0**	4	0	0
*papAH*	84 (42.9)	6	1	**8**	0	6	**18**	**10**	**7**	**6**	7
*papC*	87 (44.4)	6	1	**8**	0	7	**18**	**10**	**7**	**6**	7
*papEF*	92 (46.9)	6	1	**8**	0	**11**	**18**	**10**	**7**	**6**	7
*sfa/focDE*	58 (29.6)	1	0	**8**	0	0	**20**	0	2	**5**	**13**
*afa/draBC*	11(5.6)	7	2	0	0	0	0	0	0	0	0
*yfcV*	106 (54.1)	**20**	0	**8**	0	0	**21**	0	**10**	**7**	**14**
**Toxins**											
*sat*	47 (24.0)	13	3	0	0	9	12	0	0	0	0
*cnf1*	49 (25.0)	4	0	**8**	0	0	**17**	0	0	**7**	7
*hlyA*	55 (28.1)	3	1	**8**	0	0	**20**	0	0	**7**	7
*hlyF*	44 (22.4)	2	2	0	**4**	0	0	**8**	**7**	0	3
*cdtB*	12 (6.1)	3	0	0	0	0	6	0	2	0	0
*tsh*	7 (3.6)	0	0	0	0	0	0	0	2	0	1
*vat*	80 (40.8)	0	0	**7**	0	0	**20**	0	**10**	**7**	**14**
**Iron uptake**											
*iucD*	99 (50.5)	**16**	**6**	2	**4**	**11**	12	**8**	**7**	0	3
*iutA*	99 (50.5)	**16**	**6**	2	**4**	**11**	12	**8**	**7**	0	3
*iroN*	93 (47.4)	3	2	**8**	**4**	0	**20**	**8**	**6**	4	**14**
*fyuA*	**162 (82.7)**	**23**	**7**	**8**	**4**	**12**	**21**	**10**	**10**	**7**	**14**
*chuA*	**135 (68.9)**	**23**	0	**8**	0	**12**	**21**	0	**10**	**7**	**14**
**Capsule**											
*kpsM II*	**119 (60.7)**	**17**	2	4	0	**10**	**20**	0	**10**	**7**	**14**
*neuC-K1*	35 (17.9)	0	0	0	0	0	0	0	**10**	1	**12**
*kpsM II-K2*	17 (8.7)	7	0	0	0	3	0	0	0	0	0
*kpsM II-K5*	67 (34.2)	10	2	4	0	7	**20**	0	0	**6**	2
*kpsM III*	6 (3.1)	0	0	3	0	1	0	0	0	0	0
**Miscellaneous**											
*cvaC*	35 (17.9)	2	2	0	**4**	0	0	7	**7**	0	3
*iss*	46 (23.5)	2	2	1	**4**	0	2	**8**	5	0	5
*traT*	111 (56.6)	**17**	4	**6**	**5**	7	4	**9**	**8**	3	6
*ibeA*	23 (11.7)	4	0	0	0	0	0	0	4	0	3
*malX*	109 (55.6)	**21**	0	**8**	0	0	**21**	0	**10**	**7**	**14**
*usp*	107 (54.6)	**23**	0	**8**	0	0	**21**	0	**10**	**7**	**14**
*ompT*	**151 (77.0)**	**23**	2	**8**	**4**	**12**	**21**	**10**	**10**	**7**	**14**
**ExPEC status**	**121 (61.7)**	**15**	2	**8**	0	**9**	**21**	6	**10**	**7**	**14**
**UPEC status**	106 (54.1)	**20**	0	**8**	0	0	**21**	0	**10**	**7**	**14**
Range of VFs	1 to 23	8 to 20	1 to 14	16-19	2-10	5-12	13-20	6-13	13-22	13-17	12-23
Mean of VFs	11.6	12.3	6.3	17.1	8.4	9.4	17.4	9.8	18.2	15.4	16.2

Bold indicates the VF-encoding gene present in ≥60% of the isolates.

**Table 3:** Antimicrobial resistance observed from the 196 isolates and the isolates included in the 10 most frequent sequence types.

Drug ^a^	Number (%) of Resistant Isolates										
	Total (*n*=196)	B2 ST131 (*n*=23)	A ST10 (*n*=9)	B2 ST12 (*n*=8)	B1 ST58 (*n*=5)	D ST69 (*n*=13)	B2 ST73 (*n*=21)	C ST88 (*n*=12)	B2 ST95 (*n*=10)	B2 ST127 (*n*=7)	B2 ST141 (*n*=14)
Ampicillin AM10	**111** **(56.6)^b^**	**19** **(82.6)**	**4** **(44.4)**	**7** **(87.5)**	**5** **(100)**	**8** **(61.5)**	**6** **(28.6)**	**12** **(100)**	**3** **(30)**	**4** **(57.1)**	**3** **(21.4)**
Amoxicillin-Clavulanate AMC30	27 (13.8)	**7** **(30.4)**	0	**2** **(25%)**	0	1 (7.7)	1 (4.8)	**9** **(75)**	0	0	0
Cefazolin CZ30	27 (13.8)	**13** **(56.5)**	0	0	1 (20)	0	0	**3** **(25)**	0	0	0
Cefuroxime CXM30	21 (10.7)	**7** **(30.4)**	1 (11.1)	0	1 (20)	0	0	**3** **(25)**	0	0	0
Cefotaxime CTX30	15 (7.7)	**6** **(26.1)**	0	0	1 (20)	0	0	0	0	0	0
Ceftazidime CAZ30	3 (1.5)	1 (4.3)	0	0	0	0	0	0	0	0	0
Cefepime FED30	9 (4.6)	2 (8.7)	0	0	1 (20)	0	0	1 (8.3)	0	0	0
Cefoxitin FOX30	7 (3.6)	2 (8.7)	0	0	0	0	0	1 (8.3)	0	0	0
Aztreonam ATM30	9 (4.6)	2 (8.7)	0	0	0	0	0	1 (8.3)	0	0	0
Gentamicin GM10	17 (3.6)	4 (17.4)	0	0	0	1 (7.7)	0	**5** **(41.7)**	0	0	0
Tobramycin NN10	13 (6.6)	2 (8.7)	0	0	0	1 (7.7)	0	**5** **(41.7)**	0	0	0
Doxycycline D30	**60** **(30.6)**	**6** **(26.1)**	**3** **(33.3)**	1 (12.5)	**3** **(60)**	**5 (38.5)**	4 (19)	**10** **(83.3)**	1 (10)	**3** **(42.9)**	0
Chloramphenicol C30	23 (11.7)	1 (4.3)	1 (11.1)	1 (12.5)	0	1 (7.7)	2 (9.5)	**9** **(75)**	0	1 (14.3)	0
Nitrofurantoin FD300	2 (1.0)	0	0	0	0	0	0	0	0	0	0
Nalidixic Acid NAL30	**65** **(33.2)**	**17** **(73.9)**	**2** **(22.2)**	**3** **(37.5)**	**3** **(60)**	**7** **(53.8)**	0	**9** **(75)**	2 (20)	0	0
Ciprofloxacin CIP5	**42** **(21.4)**	**13** **(56.5)**	1 (11.1)	0	1 (20)	0	0	**9** **(75)**	1 (10)	0	0
Trimethoprim-Sulfamethoxazole SXT25	**57** **(29.1)**	**9** **(39.1)**	**2** **(22.2)**	**2** **(25%)**	**3** **(60)**	**7** **(53.8)**	3 (14.3)	**9** **(75)**	1 (10)	1 (14.3)	1 (7.1)
Multidrug resistance	**73** **(37.2)**	**18** **(78.3)**	**3** **(33.3)**	1 (12.5)	**2** **(40)**	**6** **(42.6)**	3 (14.3)	**10** **(83.3)**	1 (10)	**2** **(28.6)**	0
ESBL	13 (6.6)	**5** **(21.7)**	0	0	1 (20.0)	0	0	0	0	0	0

^a^ None of the 196 isolates were resistant to the following antimicrobials: Imipenem (IMP10), amikacin (AN30), fosfomycin (FOS200) and colistin (CL10). ^b^ When the percentage is greater than 20%, it is highlighted in bold.

## Supplementary Materials

The following are available online at https://www.mdpi.com/2079-6382/9/4/161/s1, Table S1: Phylogenetic groups of the 196 studied E. coli isolates and dominant sequence types with further characterization for ST131 (clades, subclades and virotypes), Table S2: Characteristics of the 13 ESBL-producing *E. coli* isolates and Table S3: Antimicrobial resistance and virulence factor (VF)-encoding genes.
