# Supplementary material for: Clonal Structure, Virulence Factor-encoding Genes and Antibiotic Resistance of Escherichia coli, Causing Urinary Tract Infections and Other Extraintestinal Infections in Humans in Spain and France during 2016
Source: Antibiotics (Basel). 2020 Apr 4;9(4):161. doi: 10.3390/antibiotics9040161 (PMC7235800; doi:10.3390/antibiotics9040161)
Supplement: Supplementary file 1 [file antibiotics-09-00161-s001.docx]

**Table S1.** Phylogenetic groups of the 196 studied *E. coli* isolates and dominant sequence types with further characterization for ST131 (clades, subclades and virotypes).

| Characteristic | No. (%) of isolates | | P value |
| --- | --- | --- | --- |
|  | Lugo, Spain  (n= 100) | Clichy, France (n= 96) |  |
| **Phylogenetic group** |  |  |  |
| A | 14 | 15 |  |
| B1 | 10 | 8 |  |
| B2 | 48 | 56 |  |
| C | 11 | 4 |  |
| D | 9 | 5 |  |
| E | 5 | 5 |  |
| F | 3 | 3 |  |
| **ST131** |  |  |  |
| Total | 12 | 11 |  |
| Clade A | 5 | 1 |  |
| Clade B | 2 | 2 |  |
| Cluster C1-M27 | 0 | 1 |  |
| Non-C1-M27 subclade C1 | 3 | 3 |  |
| Clade C2 (*H*30-Rx) | 2 | 3 |  |
| Clade-NT^a^ | 0 | 1 |  |
| Clonotype CH40-22 | 1 | 2 |  |
| Clonotype CH40-30 | 5 | 8 |  |
| Clonotype CH40-41 | 5 | 1 |  |
| Clonotype CH40-298 | 1 | 0 |  |
| Virotype A | 3 | 0 |  |
| Virotype A-like | 1 | 0 |  |
| Virotype C2 | 1 | 2 |  |
| Virotype C3 | 3 | 0 |  |
| Virotype D2 | 1 | 1 |  |
| Virotype D3 | 0 | 1 |  |
| Virotype D5-like | 1 | 0 |  |
| Virotype E | 0 | 2 |  |
| Virotype F | 0 | 1 |  |
| Virotype NT | 2 | 4 |  |
| **Non-ST131 ST**^b^ |  |  |  |
| ST10 | 3 | 6 |  |
| ST12 | 4 | 4 |  |
| ST58 | 4 | 1 |  |
| ST69 | 8 | 5 |  |
| ST73 | 9 | 12 |  |
| ST88 | 9 | 3 |  |
| ST95 | 6 | 4 |  |
| ST127 | 3 | 4 |  |
| ST141 | 3 | 11 | 0.0265 |

^a^ NT = Not typeable; ^b^ Represented by at least 5 isolates.

**Table S2.** Characteristics of the 13 ESBL-producing *E. coli* isolates.

| Isolate | Country | ESBL enzyme | Clone | Serotype | ExPEC  status | UPEC  status | ST131 clade, subclade and cluster |
| --- | --- | --- | --- | --- | --- | --- | --- |
| A24 | Spain | CTX-M-1 | A-CH11-54-ST34 | O9:H1 | - | - |  |
| C6 | France | CTX-M-1 | A-CH11-43-ST3596 | O8:HNM | - | - |  |
| A46 | Spain | CTX-M-1 | A-CH27-23-ST new 2-437like | O98:HNM | - | - |  |
| A7 | Spain | CTX-M-14 | B1-CH4-27-ST58 | O9:H25 | - | - |  |
| A10 | Spain | CTX-M-14 | B1-CH6-35-ST448 | O11:H10 | - | - |  |
| C22 | France | CTX-M-15 | B2-CH40-30-ST131 | O25:H4 | + | + | C2 |
| C27 | France | CTX-M-15 | B2-CH40-30-ST131 | O25:H4 | - | + | NT^a^ |
| C44 | France | CTX-M-15 | B2-CH40-30-ST131 | O25:H4 | + | + | C2 |
| C3 | France | CTX-M-27 | B2-CH40-30-ST131 | O25:H4 | + | + | C1-M27 |
| A82 | Spain | CTX-M-1 | B2-CH40-41-ST131 | ONT:H5 | + | - | A |
| C80 | France | CTX-M-55 | B2-CH14-64-ST1193 | O75:HNM | + | + |  |
| A62 | Spain | CTX-M-14 | E-CH37-27-ST405 | O18:H6 | - | - |  |
| C93 | France | CTX-M-32 | F-CH4-58-ST648 | ONT:H42 | + | - |  |

^a^ NT = Not typeable

**Table S3.** Antimicrobial resistance and virulence factor (VF)-encoding genes.

|  | Number (%) isolates | | P value |
| --- | --- | --- | --- |
| VF gene | MDR  (n=73) | non-MDR  (n= 123) | MDR vs.  non-MDR |
| Adhesins |  |  |  |
| *fimH* | 71 (97.3) | 122 (99.2) |  |
| *fimAv_MT78_* | 10 (13.7) | 17 (13.8) |  |
| *papAH* | 24 (32.9) | **60 (48.8)**^a^ | 0.03661 |
| *papC* | 26 (35.6) | **61 (49.6)** | 0.07414 |
| *papEF* | 29 (39.7) | 63 (51.2) |  |
| *sfa/focDE* | 7 (29.6) | **51 (41.5)** | 0.000001 |
| *afa/draBC* | 7 (9.6) | 4 (3.3) |  |
| *yfcV* | 28 (38.4) | **78 (63.4)** | 0.00104 |
| Toxins |  |  |  |
| *sat* | 20 (27.4) | 27 (22.0) |  |
| *cnf1* | 8 (11.0) | **41 (33.3)** | 0.00054 |
| *hlyA* | 7 (9.6) | **48 (39.0)** | 0.000014 |
| *hlyF* | 20 (27.4) | 24 (19.5) |  |
| *cdtB* | 3 (4.1) | 9 (7.3) |  |
| *tsh* | 2 (2.7) | 5 (4.1) |  |
| *vat* | 12 (16.4) | **68 (55.3)** | 8.5x10^-8^ |
| Iron uptake |  |  |  |
| *iucD* | 42 (57.5) | 57 (46.3) |  |
| *iutA* | 42 (57.5) | 57 (46.3) |  |
| *iroN* | 24 (32.9) | **69 (56.1)** | 0.00190 |
| *fyuA* | 59 (80.8) | 103 (83.7) |  |
| *chuA* | 43 (58.9) | **92 (74.8)** | 0.02546 |
| Capsule |  |  |  |
| *kpsM II* | 34 (46.6) | **85 (69.1)** | 0.00241 |
| *neuC-K1* | 5 (6.8) | **30 (24.4)** | 0.00180 |
| *kpsM II-K2* | 6 (8.2) | 11 (8.9) |  |
| *kpsM II-K5* | 23 (31.5) | 44 (35.8) |  |
| *kpsM III* | 2 (2.7) | 4 (3.3) |  |
| Miscellaneous |  |  |  |
| *cvaC* | 13 (17.8) | 22 (17.9) |  |
| *iss* | 21 (28.8) | 25 (20.3) |  |
| *traT* | **51 (69.9)** | 60 (48.8) | 0.00463 |
| *ibeA* | 4 (5.5) | **19 (15.4)** | 0.03993 |
| *malX* | 30 (41.1) | **79 (64.2)** | 0.00185 |
| *usp* | 29 (39.7) | **78 (63.4)** | 0.00176 |
| *ompT* | 50 (68.5) | **101 (82.1)** | 0.03517 |
|  |  |  |  |
| ExPEC status | 38 (52.1) | **83 (67.5)** | 0.03459 |
| UPEC status | 27 (37.0) | **79 (64.2)** | 0.00034 |
| Mean of VF | 9.90 | 12.67 |  |

^a^ Statistically significant differences are highlighted in bold.
